# Effects of Lysine on the Interfacial Bonding of Epoxy Resin Cross-Linked Soy-Based Wood Adhesive

**DOI:** 10.3390/molecules28031391

**Published:** 2023-02-01

**Authors:** Yunyi Liang, Yonghong Luo, Yang Wang, Tianyang Fei, Lili Dai, Daihui Zhang, Hongzhi Ma, Liping Cai, Changlei Xia

**Affiliations:** 1 Jiangsu Co-Innovation Center of Efficient Processing and Utilization of Forest Resources, International Innovation Center for Forest Chemicals and Materials, College of Materials Science and Engineering, Nanjing Forestry University, Nanjing 210037, China; 2 Institute of Chemical Industry of Forest Products, Chinese Academy of Forestry, Nanjing 210042, China; 3 Department of Environmental Science and Engineering, University of Science and Technology Beijing, Beijing 100083, China

**Keywords:** soy-based wood adhesive, epoxy resin cross-linking, lysine, mechanical properties, interfacial bonding

## Abstract

Soy protein isolate (SPI) is an attractive natural material for preparing wood adhesives that has found broad application. However, poor mechanical properties and unfavorable water resistance of wood composites with SPI adhesive bonds limit its more extensive utilization. The combination of lysine (Lys) with a small molecular structure as a curing agent for modified soy-based wood adhesive allows Lys to penetrate wood pores easily and can result in better mechanical strength of soy protein-based composites, leading to the formation of strong chemical bonds between the amino acid and wood interface. Scanning electron microscopy (SEM) results showed that the degree of penetration of the S/G/L-9% adhesive into the wood was significantly increased, the voids, such as ducts of wood at the bonding interface, were filled, and the interfacial bonding ability of the plywood was enhanced. Compared with the pure SPI adhesive, the corresponding wood breakage rate was boosted to 84%. The wet shear strength of the modified SPI adhesive was 0.64 MPa. When Lys and glycerol epoxy resin (GER) were added, the wet shear strength of plywood prepared by the S/G/L-9% adhesive reached 1.22 MPa, which increased by 29.8% compared with only GER (0.94 MPa). Furthermore, the resultant SPI adhesive displayed excellent thermostability. Water resistance of S/G/L-9% adhesive was further enhanced with respect to pure SPI and S/GER adhesives through curing with 9% Lys. In addition, this work provides a new and feasible strategy for the development and application of manufacturing low-cost, and renewable biobased adhesives with excellent mechanical properties, a promising alternative to traditional formaldehyde-free adhesives in the wood industry.

## 1. Introduction

The synthetic resin adhesives applied in wood product manufacturing primarily depend on fossil resources such as urea-formaldehyde resins, phenolic resins, and melamine-formaldehyde resins [1]. The market for wood adhesives is controlled by formaldehyde-based resins owing to advantages of good water resistance, short pressing times, low cost, and excellent chemical reactivity [2,3,4]. Synthetic resin adhesives are nonrenewable, and wood-based composites prepared from these adhesives release toxic substances, such as free-formaldehyde and volatile organic compounds (VOCs), which are harmful to individual health, including cancer and sensitization [5]. The combustion of these adhesives emits a significant amount of greenhouse gas CO_2_ into the atmosphere, which places the world at risk of overheating [6]. Because of the diminishing of petrochemical supply and the focus on environmental conservation, there is a growing interest in developing a low-carbon and promising alternative traditional formaldehyde-based adhesives in the wood-based panel industry [7].

Soy protein isolate (SPI) is a by-product of the soybean oil industry [8]. With the advantages of low cost, simple operation, easy access to raw materials, and biodegradable characteristics, it is an attractive natural material for fabricating wood products [9]. The SPI adhesive is commonly utilized in the wood panel industry [10]. Unfortunately, the poor mechanical performance of wood-based composites bonded with the SPI adhesive has limited its wide application [11]. Currently, two main methods could be sharpened for better performance. One is to enhance the adhesive bonding performance of the adhesive itself, such as the physical modification, chemical cross-linking modification, and biomimetic modification [12,13]. On the other hand, to enhance the bonding performance of SPI adhesives, the interfacial bonding between the adhesive and the substrate must be improved, and the adhesive’s penetration into the substrate must be enhanced by adding intermolecular lubricants and lowering the protein’s relative molecular weight [14].

Currently, chemical modification is one of the most viable modification strategies to enhance the performance of SPI adhesives [15]. The cross-linking agent can combine hydroxyl with amino groups to change the force between SPI molecules from hydrogen to covalent bonding, developing a chemical cross-linked structure [16,17]. Meanwhile, the cross-linking modification can consume the hydrophilic groups of protein molecules [18]. Commonly used cross-linking agents include epoxy compounds, isocyanates, and biomass cross-linking agents [19]. Epoxy cross-linkers interact to constitute a chemical cross-linking structure in the modification of hydrophilic groups, such as epoxy groups and amino groups, carboxyl groups [20]. Furthermore, the bonding strength of SPI adhesives is enhanced, the hydrophilic groups of protein molecules are consumed in the reaction process, and the water resistance of SPI adhesives is improved [21]. However, most cross-linking agents currently used are oily and easily soluble in water. SPI has the drawback of poor compatibility, resulting in the cross-linking agent being inhibited.

The curing of epoxy resin requires selecting a suitable curing agent [22]. Petroleum-based polyamines are commonly used as curing agents, such as dimethylenetriamine and 4,4-diaminodiphenylmethane (DDM) [23]. However, most polyamines are toxic before curing and toxicity cannot be wholly prevented, as curing agents are generally not entirely consumed and residues can be hazardous [24]. Amino acids are biobased polyamine curing agents with amino and carboxyl groups and good epoxy curing agents [25]. Lys contains two amino groups and one carboxyl group. The lone electron of the primary NH_2_ group in lysine (Lys) reacts with the epoxy resin and therefore has high reactivity [26]. Lys has a high isotropic potential (9.75), which enables it to conveniently perform cation exchange reactions. The application of Lys can reduce the potential environmental hazards of conventional amine-containing modifiers [27]. However, there are relatively few reports on curing epoxy resins with amino acids.

As a result, the water-based glycerol epoxy resin (GER) was employed as a cross-linking agent to modify the SPI adhesive chemically. Amino acids were used as accelerators to enhance the interfacial bonding with the SPI adhesive. The combination of Lys with a small molecular structure as a curing agent for soy-based wood adhesives allows Lys to penetrate easily into wood pores and can result in better mechanical strength of soy-based composites. The effect of Lys as a curing agent cross-linking on the structure and performance of SPI and its composites was investigated. This knowledge could help to manufacture green biocomposites with low cost, good mechanical properties, and thermal stability using SPI and wood.

## 2. Results and Discussion

### 2.1. Fabrication and Structural Analysis of Modified Soy-Based Wood Adhesives

**Figure 1:**
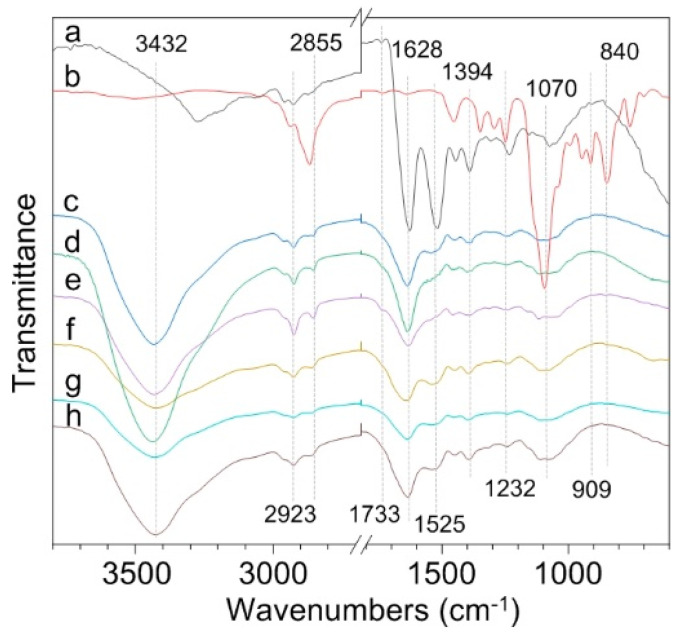
ATR-FTIR spectra of (a) SPI, (b) GER, (c) S/G-9%, (d) S/G/L-3%, (e) S/G/L-6%, (f) S/G/L-9%, (g) S/G/L-9% and (h) S/G/L-15%.

Fourier-transform infrared (FT-IR) spectra of SPI, GER, and soy-based wood adhesives are illustrated in Figure 1. The characteristic peak at 3432 cm^−1^ was related to the stretching vibration of O-H [28]. When the reaction occurred, the protein molecules became small amino acid molecules, and the GER contained O-H groups [29]. In contrast, the epoxy group and reactive groups, such as -NH_2_ and COOH, produced O-H groups that attested to the direct participation of this moiety in the cross-linking reaction. Thus, the intensity in the 3432 cm^−1^ peak increased, which can be seen from the variation between adhesives a and c–h [30]. The absorption peaks at 2923 and 2855 cm^−1^ belong to the C-H stretching bands of the saturated structural CH_2_ and CH_3_ groups, and at 1733 cm^−1^ were characteristic peaks corresponded to the C=O groups in COOH or COOR, mainly originating from amino acid small molecules [31]. The peaks of the amide groups were at 1628 cm^−1^ for the C=O stretching (amide I), at 1525 cm^−1^ for the N-H bending (amide II), and at 1394 cm^−1^ for the superposition of C-N and N-H vibrations (amide III) [32].

The peak at 1070 cm^−1^ was for the C-O-C, which mainly originated from GER and can be clearly seen in adhesive b. The reaction of GER dramatically reduced the intensity of the C-O-C absorption peak in the modified SPI adhesives c-h. [33]. The peaks at 909 and 840 cm^−1^ were attributed to the characteristic peaks of epoxy group, which was the essential characteristic peak for the hydrophilic group of GER and the SPI in the cross-linking reaction [11]. Compared with the glycerol epoxy resin b, the characteristic peaks of the epoxide groups cannot be found in the modified protein adhesive. These phenomena indicated that the epoxide groups of the GER and the -NH_2_ and -COOH groups of the protein molecule reacted sufficiently to form a cross-linking structure [20].

**Figure 2:**
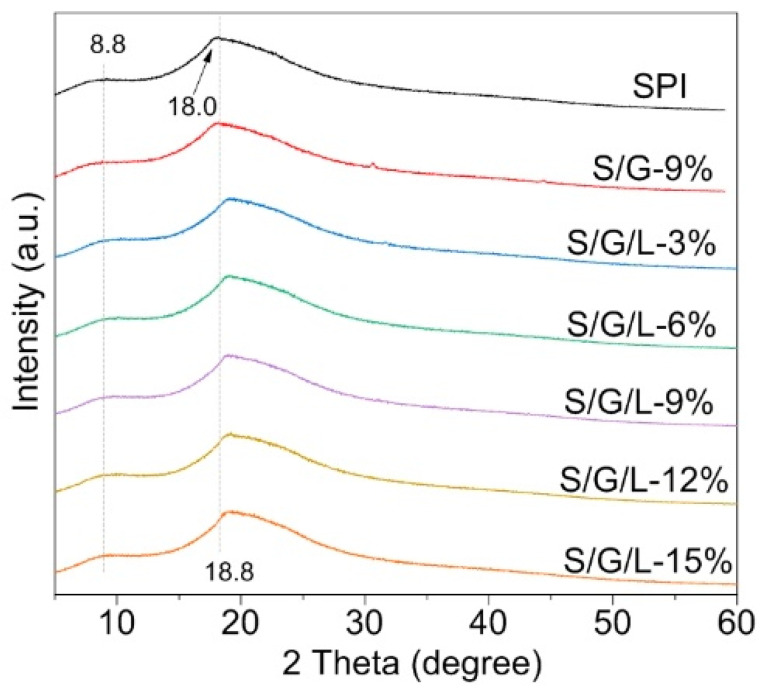
XRD patterns of SPI, S/G-9%, S/G/L-3%, S/G/L-6%, S/G/L-9%, S/G/L-12%, and S/G/L-15%.

The XRD spectra of pure SPI and modified SPI adhesives are demonstrated in Figure 2. The diffraction peaks of 2θ at 8.8° and 18.8° were the distinctive peaks of soy protein’s spiral and folded structure [34]. The diffraction angle of the folded form of the protein adhesive after the modification with the GER and Lys was slightly larger than SPI (18.0°) because the GER and Lys, as small molecules, made the lattice constant of SPI lower [35]. The hydroxyl group of GER and the amino group and carboxyl group on the adhesive agent realized a large number of hydrogen bonds to strengthen the reaction with the GER [36]. Comparing only the addition of the GER S/G-9% and the addition of GER and Lys S/G/L-3%, S/G/L-6%, S/G/L-9%, S/G/L-12% and S/G/L-15%, the diffraction angle of the folded structure increased after the addition of Lys.

Comparing SPI and S/G-9%, new diffraction peaks were observed at 30.6° and 44.3° in S/G-9%, indicating that these two peaks belong to the added glycerol epoxy. The diffraction peaks were still observed at 31.5° in S/G/L-3%, and with the addition of Lys, the diffraction peaks belonging to GER could not be directly observed. The crystallinity of the SPI adhesives is shown in Table 1. In comparison to the pure SPI adhesive, the crystallinity decreased significantly after adding GER. Moreover, the crystallinity of SPI was increased with the adding of Lys due to the limitation of the molecular chain after the formation of a cross-linked structure [20].

### 2.2. Apparent Toughness and Viscosity of SPI and Modified Soy-Based Wood Adhesives

**Figure 3:**
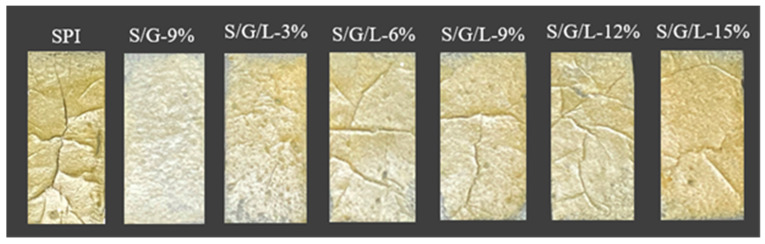
Apparent toughness of SPI, S/G-9%, S/G/L-3%, S/G/L-6%, S/G/L-9%, S/G/L-12%, and S/G/L-15%.

The apparent toughness tests of the pure SPI, adhesive modified with GER (S/G-9%), and adhesive modified with GER and Lys (S/G/L-3%, S/G/L-6%, S/G/L-9%, S/G/L-12%, and S/G/L-15%) are shown in Figure 3. The more cracks in the protein film, the worse the apparent toughness.

Compared with the pure SPI adhesive, S/G/L-9% enhanced the apparent toughness significantly after adding the cross-linking agent. The hydrophilic groups on the SPI adhesive were weakened by the interaction between the epoxy group of GER and reactive groups, including -NH_2_, COOH, and -OH [37]. The formation of the cross-linked network through the ring-opening reaction effectively improved the bonding strength. Furthermore, the water-repellent and toughening properties of GER enhanced the toughness of the modified soy-based wood adhesives. Comparing the modified soy-based wood adhesives S/G/L-3%, S/G/L-6%, S/G/L-9%, S/G/L-12%, and S/G/L-15% with the addition of GER and Lys, the apparent toughness of the modified protein adhesives decreased overall with the increase of Lys addition under the premise of fixed GER. Comparing the modified adhesives S/G/L-3%, S/G/L-6%, S/G/L-9%, S/G/L-12%, and S/G/L-15% with the addition of both GER and Lys, the apparent toughness of the modified protein adhesives decreased overall with the increase of Lys addition (Figure 3). Compared with the adhesive S/G-9% modified with the GER only, S/G/L-9% demonstrated the best adhesive strength with the addition of the GER and Lys. The addition of amino acids made the decrease of the apparent toughness of the adhesive, presumably because too much Lys instead destroyed the cross-linked structure formed by the SPI and GER, resulting in a subsequent decrease in toughness.

**Figure 4:**
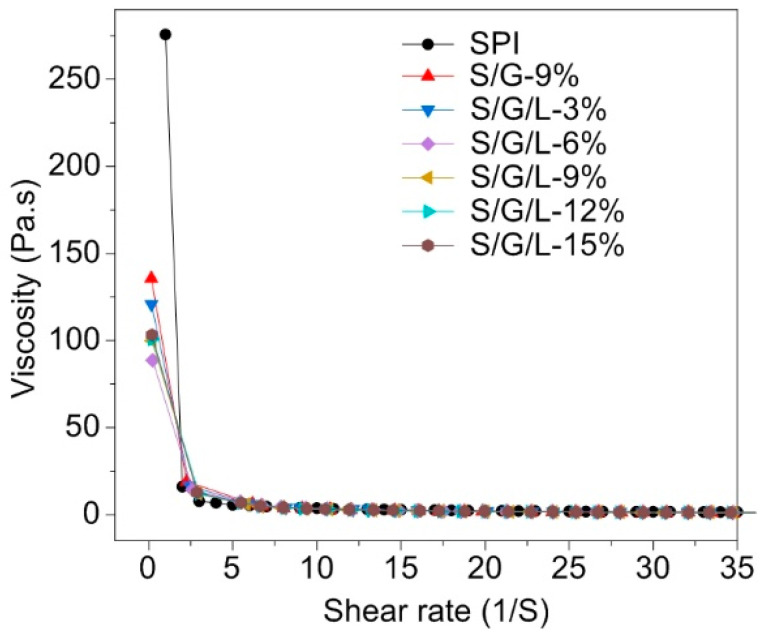
Apparent viscosity of SPI, S/G-9%, S/G/L-3%, S/G/L-6%, S/G/L-9%, S/G/L-12%, and S/G/L-15%.

Soy-based wood adhesives have the fluidity to wet and penetrate the wood [38]. The apparent viscosities of pure SPI adhesives and modified protein adhesives are displayed in Figure 4. At a constant shear rate, the apparent viscosity fell dramatically with the increase in shear time, and the difference between the initial viscosity and the subsequent viscosity was enormous. After the initial viscosity was less than 20 Pa.s, the apparent viscosity then tended to level off.

The apparent viscosity decreased considerably with the addition of glycerol epoxy S/G-9% (135.5 Pa.s) compared to the pure SPI (276.7 Pa.s) adhesive, mainly being attributed to the addition of the low-molecular-weight glycerol epoxy acting as a lubricant between the molecular protein chains, resulting in a reduction in viscosity. Compared with the pure SPI adhesive, the viscosity of the modified adhesive (S/G/L-9%) was reduced by adding 9% lysine. This phenomenon was attributed to the lubricating effect of adding a small amount of amino acid, and too much amino acid affected the viscosity of protein adhesives.

### 2.3. Micromorphology of the Modified Soy-Based Wood Adhesives

**Figure 5:**
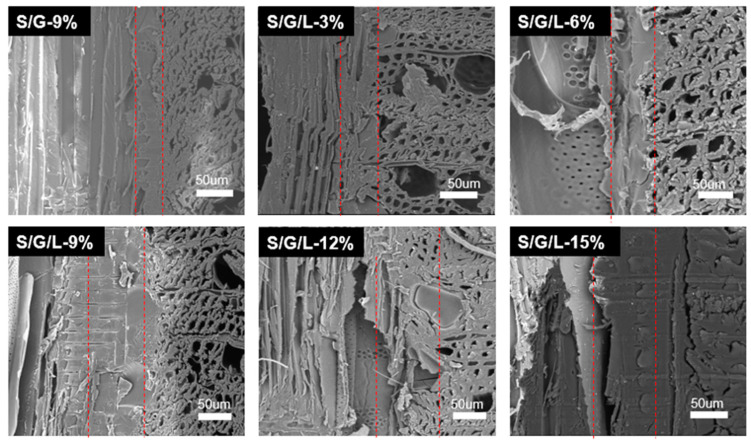
SEM images showing the penetration of adhesive in the wood substrate.

The effects of S/G-9%, S/G/L-3%, S/G/L-6%, S/G/L-9%, S/G/L-12%, and S/G/L-15% on the gluing performance of plywood were analyzed by investigating the penetration of adhesive into the substrate in the modified protein adhesives and the interfacial bonding ability of plywood (Figure 5).

In Figure 5, the red dashed area was the penetration layer’s location. Compared with S/G-9%, the modified adhesives S/G/L-6% and S/G/L-9% penetrated a significantly higher amount of wood. They filled all the voids, such as conduits of wood at the gluing interface, and the interfacial bonding ability of the plywood was enhanced. The penetration of adhesive S/G/L-9% to wood was more potent than that of S/G/L-6%, which was consistent with the best gluing performance of S/G/L-9% in the previous section.

### 2.4. Performance of the SPI and Modified Soy-Based Wood Adhesives

**Figure 6:**
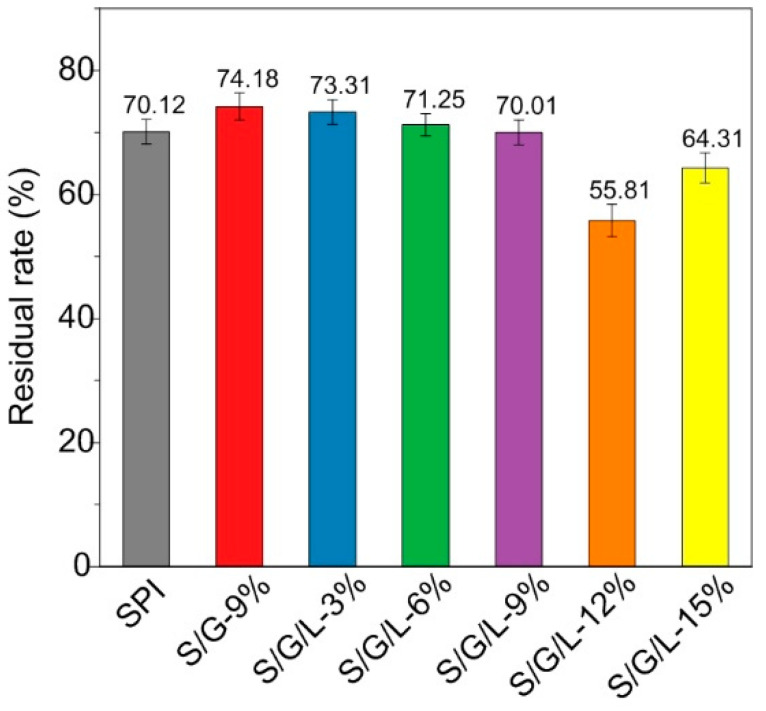
Residue rate of SPI and modified soy-based wood adhesives. The error bars represent the standard deviations of the data points in this experiment.

The residual rate is a reflection of the water resistance of the adhesive [39]. The residual rate of the adhesive S/G-9% (74.18%) modified with the GER only, and S/G/L-3% (73.31%) and S/G/L-6% (71.25%) modified with both the GER and Lys were slightly superior to the pure SPI adhesive (70.12%) (Figure 6). However, the residual rate of S/G/L-9% (70.01%) with the superior adhesive strength was identical to the pure SPI adhesive. The residual rates of S/G/L-12% and S/G/L-15% were significantly lower than those of the pure SPI in Figure 6.

In general, with the addition of the GER, the residual rate of the SPI adhesive was enhanced. This can be explained by the addition of GER consuming the hydrophilic groups in the adhesive system and developing a cross-linked network through the ring-opening cross-linking reaction, thus effectively improving the adhesive bonding strength [40]. The residual rate of the SPI adhesive decreased gradually with the addition of Lys, probably because the amino acid itself is easily tolerated in water, and the force formed by the added amino acid was quickly destroyed in water. Hence, the residual mass was decreased with the addition of Lys. The residual rate of the S/G/L-9% with the best adhesive strength was outstanding over that of the pure SPI adhesive. The residual rate of S/G/L-12% (55.81%) with the addition of Lys was significantly dissimilar from that of the pure SPI adhesive, which meant that S/G/L-9% was more in line with the requirements, which was consistent with the best adhesive strength of S/G/L-9%.

**Figure 7:**
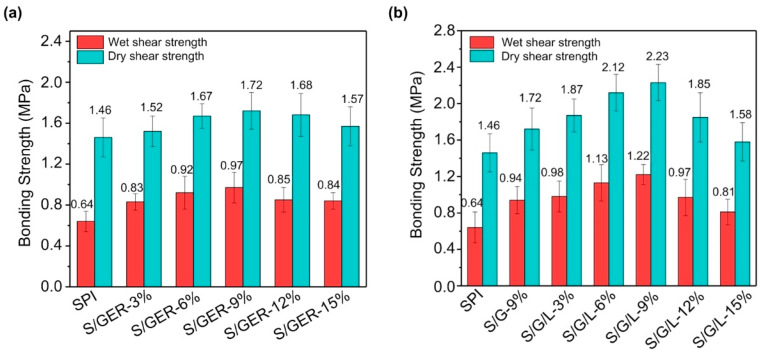
Dry and wet shear strengths of plywood prepared with the modified soy-based wood adhesives. (**a**) Bonding strength of plywood prepared by GER modified soy-based wood adhesives and (**b**) Bonding strength of plywood prepared by GER and Lys modified soy-based wood adhesives. The error bars represent the standard deviations of the data points in this experiment.

Dry and wet shear strength tests were used to assess the influence of GER and Lys on SPI adhesives (Figure 7). With the increase of the GER addition, the bonding strength of the plywood prepared with the adhesive showed an increase and then a decrease, reaching a maximum of 0.97 MPa at 9% of the GER for SPI.

The wet shear strength of the pure SPI adhesive was only 0.64 MPa, which did not meet the strength requirement of China Class II plywood (≥0.7 MPa) [41]. After adding the adhesive to glycerol epoxy and Lys, the wet shear strength of plywood climbed and then declined in comparison with the S/G/L-3%, S/G/L-6%, S/G/L-9%, S/G/L-12%, and S/G/L-15%. Compared to the pure SPI adhesive, the dry/wet shear strength of plywood prepared by the S/G/L-9% adhesive was enhanced by 52.7% and 90.6%, respectively. It was presumed that the value addition of amino acids can cause the chemical interactions between epoxy functions of the GER and reactive functions such as -NH_2_, COOH, and -OH. On the one hand, this chemical interaction decreased the content of hydrophilic groups in the adhesive. On the other hand, it formed a highly cross-linked network through the cross-linking reaction, which enhanced the adhesive bonding strength of the adhesive. When excessive amino acids were added, some amino acids did not participate in the reaction, which increased the adhesive’s solid content and destroyed the cross-linking between the GER and amino acids [42]. The wet shear strength of S/G/L-9% was suitable for the selected ratios in this study, reaching 1.22 MPa. It was indicated that the addition of Lys improved the interfacial bonding strength of the modified soy-based wood adhesives.

**Figure 8:**
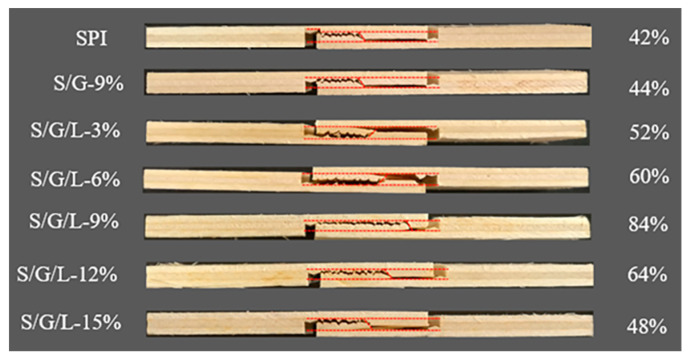
Wood failure rate of plywood prepared with SPI and modified soy-based wood adhesives.

The damage to the middle layer of plywood can reflect the adhesive bonding strength [43]. The more the part of the middle layer of wood is destroyed, the better the adhesive bonding strength [44]. The wood destruction rates of plywood prepared by the pure SPI and soy-based wood adhesives are shown in Figure 8.

The wood destruction rates of plywood prepared by modified SPI adhesives were all superior to the plywood prepared by the pure SPI adhesives, which suggested that the addition of the GER and Lys enhanced the bonding strength of SPI adhesives. The most remarkable wood destruction rate was S/G/L-9%, which reached 84%. This phenomenon illustrated that the bonding strength of the plywood climbed and then declined with the addition of Lys under the condition of a fixed amount of the GER. Comparing S/G-9% (44%) and S/G/L-9% (84%), the destruction rate of S/G/L-9% was almost twice as high as that of S/G-9%. It was revealed that adding Lys into GER significantly enhanced the bonding strength of the plywood prepared by the modified SPI adhesive.

### 2.5. Mechanical Performance of the SPI and Modified Soy-Based Wood Adhesives

**Figure 9:**
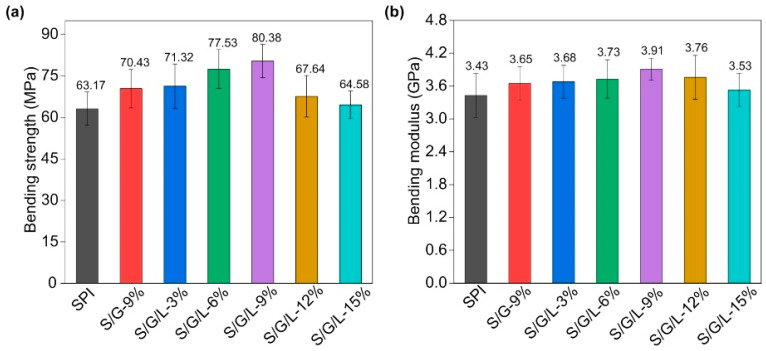
Bonding performance of plywood prepared with unmodified SPI and modified soy-based wood adhesives. (**a**) Bending strength and (**b**) bending modulus. The error bars represent the standard deviations of the data points in this experiment.

The properties of modified SPI adhesives, including flexural strength and modulus were investigated (Figure 9). Among the epoxy-based-SPI adhesives with Lys cured, the flexural modulus and flexural strength of the modified protein adhesives presented a parabolic trend with the increase in Lys addition. Compared with the protein adhesive S/G-9% modified with the GER (3.65 GPa, 70.43 MPa), the flexural modulus and flexural strength of the SPI adhesive rose by 7.2% and 14.1%, respectively, after the addition of Lys. The flexural strength and modulus of the plywood prepared by S/G/L-9% adhesive achieved 80.38 MPa and 3.91 GPa, respectively, superior to plywood prepared by pure SPI adhesives (63.17 MPa, 3.43 GPa). Compared with the SPI (3.43 GPa, 63.17 MPa), the flexural modulus and flexural strength of the SPI adhesive with adding GER and Lys increased by 13.95% and 27.2%, respectively. The results revealed that the adding of GER and Lys could enhance the gluing performance of the SPI adhesive.

### 2.6. Thermal Stability of SPI and Modified Soy-Based Wood Adhesives

**Figure 10:**
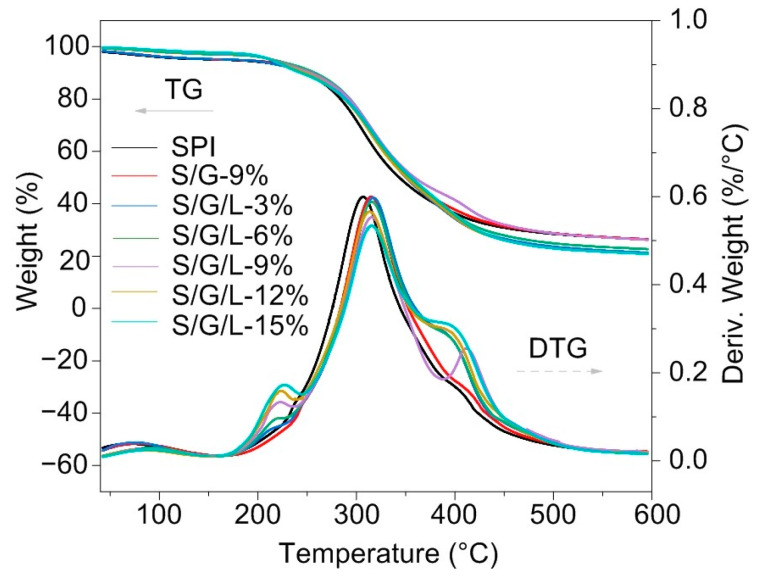
TGA and DTG curves of investigated soy-based wood adhesives.

Figure 10 demonstrates that the cross-linked SPI enhanced thermal stability, presuming that the increase in TGA mass residue was related to the insoluble content [45]. Table 2 provides the temperature ranges for the TGA mass residue at 200 °C and 600 °C as well as the maximum degradation rates for the three stages of the SPI adhesive. Combining the TG plots and the residual weight rates of protein adhesives at 200 °C, the modified soy-based wood adhesives were superior to the pure SPI adhesives. Comparing the TG curves, there was a slight rightward shift relative to the pure SPI adhesive. The thermal decomposition temperature was raised, implying that the addition of GER and Lys generated a more stable structure, boosting the thermal stability as well as the adhesive bonding performance. Furthermore, S/G/L-6% (95.1%) and S/G/L-9% (94.8%) had higher residual quality. S/G-9% (26.5%), SPI (26.3%), and S/G/L-9% (26.2%) had higher residual masses of protein adhesives at 600 °C. Comparing the residual rate at the maximum rate of the degradation in the first stage and the final residual rate, the adhesive S/G/L-9% was superior.

The DTG diagram shows that the adhesive degradation behavior comprised three reaction stages. After removing moisture in the first stage, the weak hydrogen and chemical bonds broke at 250–350 °C [46]. The peptide bonds in the main chain of SPI would be decomposed at around 350–450 °C (third stage), containing C-O, C-C, and C-N bonds [12]. The temperature at the maximum degradation rate of the third stage of S/G/L-9% (411.7 °C) was significantly higher than that of other cross-linked adhesives, which indicated that the thermal stability of S/G/L-9% was more stable than that of other modified SPI adhesives. It was indicated that the S/G/L-9% adhesive had the most complete cross-linking of epoxy and hydrophilic groups and should have the best gluing performance. This result was consistent with the most robust gluing performance of S/G/L-9% obtained in the mechanical performance.

## 3. Materials and Methods

### 3.1. Materials

The glycerin epoxy resin was obtained from Jiacheng Plastic Materials Co., Ltd. D-Lysine (98%) was purchased from Aladdin Chemical Reagent Co., Ltd. (Shanghai, China). SPI with a content of 90% was supplied by Chang Jing Sheng Wu Gongcheng Co., Ltd. (Jining, China). Poplar *(Populus* spp.) veneers (approximately a thickness of 1.5 mm, moisture content of 8.0%) were purchased from ShunChuang Material Technology Co., Ltd. (Linyi, China).

### 3.2. Preparation of the Modified Soy-Based Wood Adhesives

Firstly, 6.0 g SPI and 0.54 g GER were dispersed in 44 g DI water and mixed at 25 °C for 20 min. After adding Lys, the mixed solution was further stirred at 25 °C for 20 min to obtain the modified SPI adhesive. Table 3 lists the formulae for fabricating adhesive compounds.

### 3.3. Characterizations

Different adhesives were solidified in a blast oven at 120 ± 2 °C for the Fourier transform infrared spectroscopy (FT-IR) (ThermoFisher Scientific, Waltham, MA, USA) analysis. The scanning range was 4000–400 cm^−1^ with 32 scans. The adhesives were pre-ground into powder form, and the crystallinity was analyzed using the X-ray diffraction (XRD) (RIGAKU, Akishima-shi, Tokyo, Japan) analysis, with the diffraction angle scan range from 5 to 60°. The microstructural morphology of the adhesive interface of the plywood was sprayed with gold and photographed with a scanning electron microscope (SEM) (FEI Company, Hillsboro, OR, USA) under an accelerating voltage of 10 kV.

### 3.4. Cracking Observation

Cracks in the cured adhesives were measured to determine the toughness of the adhesives. Adhesive samples were uniformly coated on glass slides and cured for 2 h in an oven at 120 °C. The dried samples were recorded the appearance the cracks using a camera.

### 3.5. Thermal Performance

The thermogravimetric (TGA) (TA instruments, New Castle, DE, USA) analysis characterized the thermal performance of SPI adhesives. The test was taken under N_2_ atmosphere with a flow rate of 10 °C/min in the range of 30–600 °C.

### 3.6. Mechanical Performance

The mechanical performance of plywood was conducted on a universal testing machine (Shimadzu AGS-X, Nakagyo-ku, Kyoto, Japan). The sample size was 100 × 25 mm (length × width), and the loading speed was 20 mm/min. The testing distance between the two fixtures was 50 mm, and the average value was obtained by examining 13 samples.

### 3.7. Plywood Preparation and Measurement

**Scheme 1:**
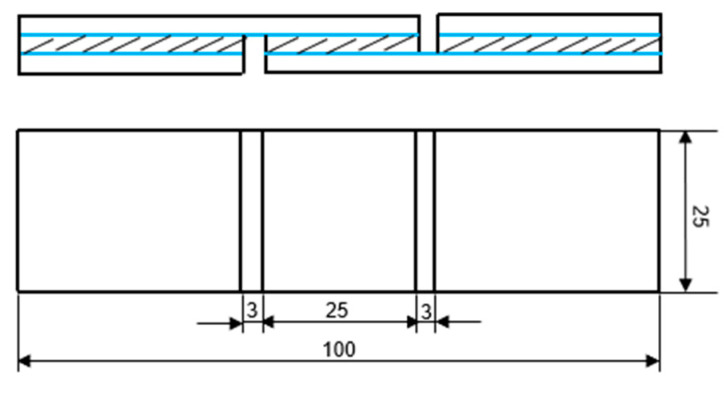
Dimensions of the three-ply plywood sample.

Three-lay plywood was fabricated to assess the bonding performance of these adhesive compositions. The adhesive of various formulations was evenly coated on one side of the 1.5 mm–thick poplar veneer with a density of 200 g/m^2^. The uncoated veneers were stacked between two veneers coated with adhesive according to the principle of vertical texture between adjacent veneers. Then, the adhesive-coated veneers were placed in a cold press after 10 min for prepressing. Subsequently, the plywood was pressed at 120 °C and 1.0 MPa for 5.5 min using a hot press (Qiulin Machinery, China). Then, the shear strength was assessed according to the Chinese national standard (GB/T 17657-2013) [47]. The dry shear strength of the plywood samples was tested on a universal testing machine. Plywood samples were soaked in water at 63 ± 2 °C for 3 h before being cooled at room temperature for ten minutes to determine the wet shear strength. Scheme 1 depicts the dimensions of the plywood specimens.

### 3.8. Apparent Viscosity Measurement

The apparent viscosity of adhesives from 0 to 150 s^−1^ in increments of 10 s^−1^ at 25 °C was examined with a Brookfield rotational viscometer (Middleboro, MA, USA).

### 3.9. Residual Rate Testing

The SPI adhesives were placed at 120 ± 2 °C, and the masses were recorded as *M*_1_ at constant weights, the samples were soaked in water at 60 ± 2 °C for 6 h, then dried at 105 ± 2 °C. Again, the constant weights were recorded as *M*_2_, and the residual rate was calculated as:

(1) $$\mathrm{Residual}\;\mathrm{rate}\;\left(\%\right)=\frac{M_{2}}{M_{1}}\times 100$$

## 4. Conclusions

The preparation of modified soy-based wood adhesives with Lys as a curing agent improved the cross-linking reaction activity and physical penetration of soy protein into the wood, thus effectively enhancing the interfacial adhesion between the soy protein adhesives and veneers. Furthermore, the Lys has the same amine group as petroleum-based curing agents, and is an eco-friendly alternative to agent epoxy resin curing in the SPI cross-linking agents. The modified SPI adhesives had enhanced mechanical properties, apparent toughness, and thermal stability. The modified SPI adhesive manifested that the pyrolysis temperature was increased, and the thermal stability was improved. Compared to the pure SPI adhesive, the residual weight of S/G/L-9% was 26.2 wt% at degradation temperatures up to 600 °C. Compared to the pure SPI adhesive, the dry/wet shear strength of plywood prepared by the S/G/L-9% adhesive was enhanced by 52.7% and 90.6%, respectively. The flexural strength and modulus of the plywood prepared by S/G/L-9% adhesive achieved 80.38 MPa and 3.91 GPa, respectively, superior to plywood prepared by SPI adhesives (63.17 MPa, 3.43 GPa). Such Lys cross-linking greatly improved the dry/wet shear strength, thermal stability, and water resistance. Overall, this study avoids the environmental pollution caused by the extensive use of petroleum-based curing agents in adhesives. The effect of Lys cross-linking as a curing agent on the structure and properties of SPI and its composites was investigated, providing a theoretical basis for the preparation of new low-cost and ecological adhesives as an alternative to formaldehyde-based adhesives.

## Figures and Tables

**Table 1:** Relative crystallinity of SPI and modified soy-based wood adhesives.

Sample	RCI ^a^
SPI	21.6 (0.7) ^b^
S/G-9%	15.4 (0.5)
S/G/L-3%	18.9 (0.7)
S/G/L-6%	16.3 (1.0)
S/G/L-9%	21.4 (0.4)
S/G/L-12%	20.6 (0.5)
S/G/L-15%	22.3 (0.9)

Note: ^a^ relative crystallinity, ^b^ standard deviation.

**Table 2:** Temperature of the maximum degradation rate and residue weight fraction for different cured adhesive samples.

Sample	Tpeak1 (°C) ^a^	Tpeak2 (°C) ^a^	Tpeak3 (°C) ^a^	Residue (wt.%)	
				T200 °C ^b^	T600 °C ^c^
SPI	218.1	304.1	400.5	93.2	26.3
S/G-9%	220.5	313.3	407.3	93.8	26.5
S/G/L-3%	216.7	314.2	386.2	93.4	21.3
S/G/L-6%	218.7	312.4	390.4	95.1	22.7
S/G/L-9%	220.4	314.1	411.7	94.8	26.2
S/G/L-12%	222.6	312.3	393.8	94.5	20.3
S/G/L-15%	223.8	313.4	392.2	94.2	20.9

Note: ^a^ Temperature corresponding to the maximum degradation rate of the three stages; ^b^ residual mass of adhesive at 200 °C; ^c^ residual mass of adhesive at 600 °C.

**Table 3:** SPI adhesive formulations.

Sample	DI Water (g)	SPI (g)	GER (g)	Lys (g)
SPI	44	6	-	-
S/GER-3%	44	6	0.18	-
S/GER-6%	44	6	0.36	-
S/GER-9%	44	6	0.54	-
S/GER-12%	44	6	0.72	-
S/GER-15%	44	6	0.90	-
S/G-9%	44	5.46	0.54	-
S/G-9%/L-3%	44	5.82	0.54	0.18
S/G-9%/L-6%	44	5.64	0.54	0.36
S/G-9%/L-9%	44	5.46	0.54	0.54
S/G-9%/L-12%	44	5.28	0.54	0.72
S/G-9%/L-15%	44	5.10	0.54	0.90

Note: S/G-9%/L-3% means the total mass of SPI and Lys is 6 g, where the mass of amino acid is 3% of the total, the mass of GER is 9% of the total mass of SPI and Lys, and the others are analogous.

## Data Availability

The data presented in this study are available in the article.
